# Review of the Performance of High-Voltage Composite Insulators

**DOI:** 10.3390/polym14030431

**Published:** 2022-01-21

**Authors:** Muhammad Zaheer Saleem, Mohammad Akbar

**Affiliations:** Faculty of Electrical Engineering, Ghulam Ishaq Khan Institute of Engineering Sciences and Technology, Topi, Swabi 23460, Pakistan; akbar@giki.edu.pk

**Keywords:** SiR, EPDM, epoxy, micro/nano fillers, material degradation, polymeric insulators

## Abstract

In the present literature survey, we focused on the performance of polymeric materials encompassing silicone rubber (SiR), ethylene propylene diene monomer (EPDM) and epoxy resins loaded with micro, nano, and micro/nano hybrid fillers. These insulators are termed as composite insulators. The scope of the added fillers/additives was limited to the synthetic inorganic family. Special attention was directed to understanding the effect of fillers on the improvement of the thermal conductivity, dielectric strength, mechanical strength, corona discharge resistance, and tracking and erosion resistance performance of polymeric materials for use as high-voltage transmission line insulators. The survey showed that synthetic inorganic fillers, which include silica (SiO_2_) and hexagonal boron nitride (h-BN), are potential fillers to improve insulation performance of high-voltage insulators. Furthermore, nano and micro/nano filled composites performed better due to the better interaction between the filler and polymer matrix as compared to their only micro- or nano filled counterparts. Finally, some aspects requiring future work to further exploit fillers are identified and discussed.

## 1. Introduction

In overhead transmission systems, insulators provide necessary insulation between each phase and grounded crossarm to prevent the leakage of current to earth as well as to support line conductors. This demands that insulators should possess high electrical insulation resistance, a high mechanical strength-to-weight ratio [1], high thermal conductivity, easy molding and lower maintenance cost [2]. Because of the nature of the duties, the selection of insulators depends both on the economic consideration and assurance of long-term performance [3,4].

Historically, traditional porcelain and glass insulators have been used extensively as outdoor high-voltage insulation, and their required material properties have been known for a long time [5]. Decades of in-service experience illustrates that these materials possess excellent resistance to electrical and environmental stresses and, at the same time, are cost effective [6]. However, from a utility perspective, their labor-intensive installation and maintenance complexities due to their bulkiness and poor performance in highly polluted areas are the associated concerns and challenges associated with these materials [7].

Polymeric insulators, with their advent in 1960 [8], were presented as a potentially attractive alternative to ceramics for use by electric utilities and equipment manufacturers. A polymeric material, by virtue of its chemical formulation, offers greater ease in creating complex designs, as well as fabrication. The substantial advantages which a polymeric insulator offers include light weight [9,10], a compact line design and mitigation against consecutive failures. Furthermore, its low surface energy means that the polymeric insulator surface’s hydrophobicity remains intact even under wet conditions [11,12,13]. The light weight of a polymeric insulator has an impact on the economic design of towers, thus making the upgradation of transmission voltage possible without any significant dimensional changes to supporting towers [14].

The commonly used polymeric materials for high-voltage outdoor insulators are silicone rubber (SiR), ethylene propylene diene monomer (EPDM), epoxy resins and ethylene vinyl acetate (EVA) [4,9,15]. These materials are used to manufacture sheds which offer required creepage distance, while the mechanical strength is provided by the fiberglass rod. Some other polymeric materials such as polytetrafluoroethylene (PTFE), polyolefin elastomers (POE) and high-density polyethylene (HDPE) find their applications at a low voltage level both in outdoor and indoor environments [16]. The polymeric materials (SiR, EPDM, and epoxy resins) are classified as thermoset elastomers, while the remainder are thermoplastic polymers. The thermoset elastomers have been extensively studied for manufacturing sheds of the first generation of polymeric insulators [17]. In the early 1960s, outdoor insulators manufactured from epoxy resins were used for the first time in the UK. However, surface damage and poor performance at cold temperatures were the main constraints leading to their failure in outdoor environments [16]. Later, polymeric insulators were used elsewhere, and the statistics of their worldwide use are given in [18], which shows the extensive use of SiR and EPDM. The popular use of SiR as a shed material is due to its characteristics offering the ability to recover surface hydrophobicity in a harsh environment, excellent breakdown strength and high volume resistivity. However, it is less resistant to tracking, weak mechanically, and is also expensive [19,20]. On the other side, EPDM exhibits better resistance to tracking/erosion and is relatively stronger mechanically. However, it has low surface and volume resistivity as compared to SiR [21,22]. Considering the overall performance, polymeric insulators made of SiR were reported to have a relatively superior standing [23,24,25].

The major concern associated with polymeric insulators is their life expectancy. Being organic in nature, SiR, EPDM, and epoxy in their pure forms are bound to age during their extended exposure to the outdoor environment. This results in the partial degradation of their dielectric, thermal and mechanical properties [16,26]. Under electrical and environment stresses in real working environments, polymeric sheds experience degradation which is weakly bonded at the molecular level. Electrical stresses, which include corona discharges and dry band arcing, along the highly stressed surface regions cause surface tracking/erosion and finally cause the puncturing of the sheds [27]. The environmental stresses which contribute to the aging of polymeric insulators are dry sunlight heating in arid areas, ultraviolet (UV) radiation, moisture, and acid rain, etc. [27,28]. These environmental parameters may lead to a thermal impact due to heating and radiation, the corrosion of metal-end-fittings and the flow of leakage current on the degraded surface under moist conditions. It may cause flashovers and ultimately failure of the sheds, leading to permanent failure of the insulators. Having understood the failure mechanisms of the first generation of polymeric insulators under prevailing electrical and environmental stresses, researchers diverted their attention to modify these polymeric insulator materials through the incorporation of micro and nano fillers.

The polymeric insulators of the second generation are fabricated using different polymeric materials loaded with different fillers. Researchers have studied and analyzed the thermal, dielectric, and mechanical performance of SiR, EPDM, and epoxy filled with micro, nano, and micro/nano hybrid fillers to achieve enhanced electrical, thermal and mechanical properties. Accordingly, in this literature review, our focus is to discuss the performance of polymeric materials filled with different inorganic materials. The studies conducted at a global level are reviewed, encompassing composites which have shown some potential to manufacture the second generation of polymeric insulators. This survey also summarizes the impact of inorganic fillers on important age-retarding phenomena such as corona-caused aging, tracking and erosion resistance, and hydrophobicity recovery performance. Various proposed mechanisms responsible for enhancing performance are deliberated upon, and areas requiring future research to further enhance the desirable properties of composite insulators are discussed.

## 2. Composite Polymer and Fillers

A composite is defined as a material system typically composed of two or more constituents that are different in shape/chemical composition, retain their physical identities and perform functions different from their individual components. One of the composite materials which are used in bulk quantity is usually a polymer known as the base material, while other constituents include one or more additives/fillers. In the case of two or more additives/fillers or base materials, the material system is designated as a hybrid composite. In electrical insulation systems, the term filler is used rather than additive [29]. For high-voltage insulation systems, the most commonly used synthetic inorganic fillers are from the families of oxides (silicon dioxide or silica (SiO_2_), titanium oxide (TiO_2_), aluminum oxide (Al_2_O_3_), zinc oxide (ZnO), magnesium oxide (MgO)), nitrides (boron nitride (BN), aluminum nitride (AlN), silicon nitride (Si_4_N_3_)) and carbides (silicon carbide (SiC)). These fillers have been used to enhance the desirable properties of polymeric materials [23].

While studying polymeric insulators involving a variety of fillers, the homogenous dispersion of fillers in the polymeric material is very important to avoid any cluster formation. This is due to their interfaical tension and to provide improved adhesion between the filler and the polymer. This can be achieved by means of the surface modification of added filler(s) into the polymeric matrix [30,31,32]. The interface between the filler and the polymeric base material plays a significant role. It has been reported that better dispersion improves compatibility between a polymeric material and the filler surface to avoid agglomeration [33]. This can be achieved by adding coupling agents such as titanate (TC9), 3 methaacryloyloxy propyltrimethoxysilane (KH570) or γ-2, 3 epoxypropoxy-propytrimethoxysilane (KH560) to obtain surface modification of the filler [32,33,34,35]. For surface modification of a filler, its surface is functionalized with one of the abovementioned coupling agents. The following chemical reactions take place by means of the addition of a silane coupling agent: (a) hydrolysis; silanol is produced from alkoxy group of silanes and becomes attached on the filler surface; (b) when mixing the functional filler with the polymeric base material, a reaction takes place between the polymeric material and organic groups of the coupling agent attached onto the surface of the filler. After curing and the final preparation of sample, the filler dispersion was reportedly checked using a scanning electron microscope, which confirmed that filler surface modification reduces the agglomeration and hence better dispersion can be achieved [35].

## 3. Role of Fillers on Important Properties of Polymeric Insulators

### 3.1. Enhancement of Thermal Conductivity

One of the desirable properties of an insulating material is its high thermal conductivity. As most polymeric materials (including outdoor high-voltage insulators) have a thermal conductivity lower than 0.5 W/m·K (see Table 1), this may result in their easy degradation at higher temperatures due to heat build-up during dry band arcing. It can, however, be enhanced by means of two methods. The first method is to use an intrinsic thermal conductive polymer which offers higher thermal conductivity due to its highly ordered molecular chain structure [36]. However, due to their high cost and complex manufacturing process, the use of intrinsic thermal conductive polymers has limited applications, particularly in electrical equipment. The other method is to make a composite of a polymer by adding a filler of higher conductivity, such as BN, AlN and Si_3_N_4_, as summarized in Table 1. It is, however, difficult to compare the thermal conductivity of different composites due to variations in filler size, its content, shape, orientation, surface treatment and method of composite manufacturing. In the following paragraph, an attempt is made to explain the general trends and mechanisms which could affect the thermal conductivity of the composite.

During the past few years, research efforts have prominently been made to enhance the thermal conductivity of polymers by adding fillers of varying size, ranging from micro to nano, as well as their hybrids. The enhanced thermal conductivity of silicone rubber filled with micro ATH, AlN and h-BN was highlighted in [37]. The gain of thermal conductivity was about 2.6, 2.3 and 6.8 times higher for 30 wt% each of ATH, AlN and h-BN, respectively, as compared to their unfilled counterparts. The highest enhancement of thermal conductivity of h-BN filled silicone rubber has been attributed to better cross-link points between h-BN hydrophilic OH group and Si-o-Si functional groups of the main chain. Another study [43] conducted on epoxy filled with equal concentrations of nano-AlN and nano-MgO exhibited almost the same enhancement of thermal conductivity, although the added fillers had significantly different thermal conductivities. From these results, firstly, it was demonstrated that the thermal conductivity of base polymer increases by adding a filler. However, a non-linear rise of thermal conductivity was noticed by considering the thermal conductivity of fillers. This behavior suggests that a filler with higher thermal conductivity may not always lead to higher thermal conductivity of the composite. It may be conjectured that the filler’s interaction with the base polymer is another important parameter affecting thermal conductivity. Secondly, in the case of a micron-sized filler, a large volume is required to improve the thermal conductivity of the composite. This behavior suggests that at a low volume/mass fraction of the filler, thermal conductive chains are not formed effectively in the composite. However, incorporation of a micro filler at 30 wt% or more plays a significant role in enhancing the thermal conductivity of the polymer material (also confirmed in Table 2).

Heid and co-workers studied the impact of filler size on thermal properties of epoxy filled with micro and submicro particles of h-BN [50]. At equal wt% of each filler, submicro particles caused 6% more enhancement of thermal conductivity as compared to micron-sized particles. By decreasing the filler size from micro to nano, the thermal conductivity increases with the increase in filler content [53]. Similarly, a filler with a high aspect ratio (i.e., ratio of the maximum to the minimum dimensions of the filler) also helps to form a composite of high thermal conductivity [52]. These observations lead us to conclude that by increasing the filler volume with filler particles of a larger size, thermal conductivity decreases due to increased porosity [39]. Through this observation, although thermal conductivity of composites improved though varying filler types, it was not crucially valuable as compared to the size of fillers to enhance the composite thermal conductivity.

Nazir and co-workers reported that thermal conductivity can be increased by adding only a small number of nano-sized particles in a composite filled with micro-sized particles. For example, thermal conductivity of 29 wt% of 5 µm BN + 1 wt% of 50 nm BN-filled EPDM composite was about 18% higher as compared with its counterpart filled with 30 wt% of micro-BN only [54]. The effect of volume fraction of nano-sized filler incorporated in a micro filled polymeric composite was also investigated in [51]. The results show that 26 wt% of 0.3 to 3 µm Si_3_N_4_ + 4 wt% of 20 nm Al_2_O_3_-filled silicone rubber composite exhibited the highest thermal conductivity. These studies suggest that a micro/nano hybrid filler is more effective in enhancing thermal conductivity even with a lower percentage of nanoparticles than only a micro filled composite. It appears that the addition of nano-sized particles even with a low concentration fill the gaps between micro-sized filled polymeric composite and thus the facilitate formation of easier thermal conductive pathways resulting in increased thermal conductivity.

### 3.2. Enhancement of Electrical Properties

In this section, a well-known process leading to the breakdown of a polluted polymeric insulator is discussed. Moreover, the impact of a filler, individual or hybrid (containing more than one constituent), in the polymer affecting the process has been elaborated upon.

The distribution of the electric field on the surface of a wet/moist insulator may distort due to the flow of leakage current and the formation of dry bands, which result in random surface discharges. These discharges, depending on their severity, may damage surface hydrophobicity by consuming the thin polymeric layer and cause further enhancement of electric field intensity, leading to more surface electrical discharges. As water molecules are polar and have higher conductivity than polymeric materials, they decrease the flashover voltage due to increased polarization and dielectric loss of the material [55].

Furthermore, the pores/voids inside the bulk polymeric insulator may cause initiation of partial discharge activities and decrease the dielectric strength due to the accumulation of charge [52]. The occurrence of electrical discharges on the surface is the root cause of the surface degradation of a polymeric insulator, which ultimately leads to complete failure.

It has been reported that the dielectric strength of a base polymer can be modified by incorporating different inorganic fillers which generally enhance performance at low filler loading. This may be affected by several factors, including the type of filler, its shape, size and dispersion, as well as the properties of the matrix (base polymer) itself. However, above a certain volume/weight concentration, called the dielectric percolation threshold, the dielectric strength may decrease [56]. The percolation threshold is defined as the transition behavior of a composite dielectric with increasing filler content. The formation of dielectric percolation is based on the polymer and filler interface, which gives the peak value of dielectric strength of a composite [34]. Gao et al., for example, found the dielectric percolation threshold in nano silica/epoxy composite is 5 wt% silica [34]. Similarly, the percolation threshold of carbon black-filled composite is 6.2 wt%, as discussed by Zois and co-workers [57]. The enhancement of dielectric strength through the addition of a filler has been attributed to the induced shallow traps which retard the accumulation of space charge. However, beyond a certain wt% of filler, the percolation path is formed due to the easy overlapping of charge carriers in a double layer, thus resulting in a reduction in dielectric strength. Moreover, by increasing the filler content, the interparticle distance becomes equal to the diameter of filler particle, which provides an easy path to overlap charge carriers and dielectric strength shows a decreasing trend [58]. Frechette et al. studied and characterized the interphase and overlap regions by adding large contents of nano filler in epoxy [59]. They used a 2D computer generated composite model having random particle distribution with no interphase. The interphase volume fraction was reported to saturate at 37 nm of the interphase radius, and the remaining fraction was used as an overlap ratio by the added filler. The reported results also indicate that interphase particle overlap imparts a significant influence on the dielectric response of the composite. The addition of less than 1 wt% nano size ZnO in epoxy resin significantly increased its dielectric strength under AC voltage [46]. The experimental studies on epoxy/polyhedral oligomeric silsesquioxanes (POSS) were performed to enhance dielectric strength and thermal conductivity behavior by Heid and co-workers [60]. Andritsch et al. reported an increase in the dielectric strength of epoxy filled with nano BN, which was attributed to the surface modification of the polymer nanocomposite [61]. The increase in dielectric strength at smaller sized filler loading was attributed to the better suppression of mobile charge carriers at the interface region between the polymer matrix and filler. This restricts the enhancement of electrical stress. It appears that a further decrease in the size of the filler may increase the dielectric performance, since this offers even better penetration charge carriers through the bulk of the composite. The authors in [62,63] reported the results of dielectric strength of 9000 h-aged samples in a multi-stress environment of unfilled HTV-SiR and its composites filled with different fillers. Among these, HTV-SiR filled with 6 wt% nano SiO_2_ + 20 wt% micro ATH gave the highest AC dielectric strength. Other researchers [64,65] also found that the dielectric performance of nano and micro/nano filled composites was better as compared to their only micro filled materials. An estimated model offering higher insulation dielectric strength for micro/nano filled epoxy resin was presented in [66]. It is assumed that the addition of nano filler in micro filled composite enhances its capacity to prevent progression of treeing in the composite.

In various other studies, the impact of inherent permittivity of the filler on dielectric strength of the composites was investigated [67,68]. These studies revealed that an epoxy composite with 10 wt% nanoclay offers the highest dielectric strength of 26.21 kV/mm among various investigated fillers of Al_2_O_3_, TiO_2_ and Ca_2_O_3_. This is due to the lowest inherent permittivity of nanoclay among these fillers [68]. Similarly, 20 nm nano SiO_2_-filled epoxy was reported to offer higher dielectric strength in comparison with 40 nm nano Al_2_O_3_-filled epoxy. This was attributed to less field enhancement caused by SiO_2_ particles [69]. These studies also concluded that the difference in relative permittivity and electrical conductivity of the filler and the base polymer should be small. This helps to obtain higher dielectric strength of the composite insulators by preventing the enhancement of electric field and its distortion. Table 3 lists the increased dielectric strength of various polymeric composites by adding micro, nano and micro/nano fillers.

### 3.3. Enhancement of Mechanical Properties

In addition to electrical properties, mechanical properties are equally important for the reliable operation of a polymeric insulator. The life-time prediction of insulators at high temperature and humidity was assessed through the reduction in mechanical properties and was attributed to the oxidation and thermolysis of side chain of polymer material [1]. Researchers attempted to enhance this through the incorporation of various fillers in polymers such as SiR, EPDM, and epoxy used for manufacturing polymeric insulators.

In these studies, the effects of filler type, its size and shape, filler loading, and dispersion on the mechanical properties of polymeric composites were explored. For example, the mechanical properties of epoxy were reported to be improved through the incorporation of h-BN nano particles, and the same was the case with EPDM filled with h-BN nanoparticles [21,70]. The tensile strength of silicone rubber filled individually with spherical particles of SiO_2_ and ATH was 6.6 and 3.4 MPa, respectively [71]. The higher tensile strength of silicone rubber composite filled with SiO_2_ was attributed to the stronger interfacial interaction between SiO_2_ particles and silicone rubber as compared to ATH particles and silicone rubber. On the other hand, the irregular shape of a filler having a high particle aspect ratio limits the contents of filler (to avoid sedimentation), and thus the tensile strength may not increase much [52]. For example, it has been reported that the tensile strength of BN-filled SiR is lower as compared to its ATH-filled counterpart. This may be attributed to the high aspect ratio of BN, which becomes the source of more defects in the composite [72]. These studies suggest that the tensile strength increases due to the addition of the filler regardless of its type or shape. However, this increase mainly depends upon the degree of effectiveness of interaction between the filler particles and the polymer base material. After exposure to long-term multi-stress aging under AC and DC voltages [73,74,75], Akbar and co-workers measured the tensile strength and elongation-at-break of silicone rubber filled with micro/nanoparticles. It was found that the better interaction and dispersion of added particles (20 wt% micro + 2 wt% nano SiO_2_) in silicone rubber as well as hydrogen bonding between silanol and -OH groups of SiO_2_ and silicone rubber, respectively, were possible reasons for retaining good mechanical strength even after long-term aging [73]. With the addition of 20 wt% micro and 5 wt% nano SiO_2_, a composite of SiR (as listed in Table 4) exhibited the higher change in mechanical properties in comparison to EPDM and epoxy composites. This has been attributed to strong crosslinking and strong hydrogen bonding to cause a reduction in chain mobility. Similarly, Nazir et al. [22] studied the mechanical properties of EPDM filled with hybrid micro/nano BN filler and reported a high tensile strength of 5.2 MPa for 25 wt% micro + 5 wt% nano BN particles. Assuming the uniform dispersion of filler particles, as considered in Tanaka’s model, the decrement of inter-particle distance caused by the addition of fillers in the hybrid composite is the contributing factor to enhancing mechanical properties. These properties offer excellent resistance against both dry band arcing as well as allied heat build-up during the discharge activities, and hence tracking time increases considerably.

### 3.4. Corona Degradation Performance

Transmission lines are built to operate on a continuous and long-term basis. Corona is one of the unavoidable electrical phenomena and potential threats causing the degradation of polymeric insulators during their long-term operation. In the outdoor service environment, the presence of moisture on the insulator surface may initiate corona activities [76,77]. The gaseous products (NO and NO_2_) produced by corona may react with moisture and form HNO_3_ which could be harmful both for shed material as well as the core [78]. A corona generates electrons, ozone, and ultraviolet radiation. It also causes an audible noise, radio interference and power loss. A corona on the surface of polymeric insulators may create a silica layer (known as chalking), cracks and formation of OH hydrophilic groups due to chain auto-oxidation, and scission of the polymer chain to lower-mass molecular compounds. The silica layer so formed impacts the porosity of the material, affecting moisture absorption, which is considered as the main factor contributing to material failure due to corona discharges [78]. The increased porosity which helps the moisture ingress into silicone rubber by providing diffusion pathways also contributes in reducing its hydrophobic performance [79]. Zhang and co-workers [80] presented results indicating that the saturated water absorption ratio and the relative content of Si and O elements are significantly correlated with operating time. These factors can be used to characterize the degree of aging of composite insulators. Other secondary factors contributing to failure are the polar groups originating from the surface layer cracking. This may lead to the surface degradation of polymeric insulators [81]. Surface cracks may modify the mechanical and electrical properties of these insulators [82]. Several researchers have made attempts to understand corona-caused degradation using single and multiple-needle electrode test systems [83,84,85]. The purpose of multiple needles is to produce a large area of the degraded surface for different investigations, which is not possible otherwise by using a single-needle electrode arrangement. To better understand corona-caused surface degradation, diagnostic techniques such as scanning electron microscopy (SEM) and Fourier transform infrared spectroscopy (FTIR) were employed.

In an earlier phase of these studies, an attempt was made to understand corona-related aging due to applied electric stress. For example, in their study, Reddy and co-workers used silicone rubber with 50 wt% ATH and reported a more damaging effect of AC corona in comparison to DC corona under negative polarity [83]. Similarly, the behavior of a water droplet on epoxy resin nanocomposites under AC and DC corona discharges was studied in [89]. It was noticed that under AC voltage, the water droplet thins out in the highly stressed region and causes the initiation of the discharges from the edges of the water droplet. It was also reported that a positive DC corona has a lower tendency to impact surface oxidation as compared with AC corona [78]. Vas and Thomas [90] studied corona surface discharges of composite materials under DC voltage with specific reference to polarity effect. As an outcome, it was reported that a negative DC corona is relatively more severe in the context of surface degradation and loss of hydrophobicity as compared to a positive DC corona. These studies suggest that in the case of a positive DC corona, the charge decay is the slowest as compared to AC and negative DC, which prevents further charges from reaching the surface and thus prevents more surface degradation.

Having obtained relevant data regarding corona-caused aging from different studies, new research initiatives were undertaken to mitigate aging. To that end, studies have been undertaken involving the use of different inorganic fillers to observe their impact on the properties of polymers, such as hydrophobicity and tracking/erosion. In a study using 5 wt% nano silica filled silicone rubber, the loss of hydrophobicity was lower as compared to unfilled silicone rubber after 72 h of AC corona [91]. This damage control was attributed to the nano filler which suppressed the partial discharge activity and associated loss of hydrophobicity. Through the incorporation of nano fillers, the interparticle spacing reduced by 98.5%, with a consequent increase in total surface spacing area by ~3391% as compared to micro filled silica composite [92]. This enhanced the barrier resistance, which impedes damage of organic structure.

Various studies were also conducted to examine the effect of micro/nano hybrid filled composites on corona discharge. Tariq et al. performed AC corona discharge experiments on silicone rubber loaded with a micro ATH/nano Al_2_O_3_ hybrid filler [93]. The aim of this study was to understand the characteristics of the area directly below the electrode needle tip and in its vicinity. It was found that the area directly below needle tip offered 2.5 times more resistance to surface roughness of silicone rubber composite filled with 38 wt% micro ATH + 2 wt% nano Al_2_O_3_ in comparison to its micron-filled counterpart. Lyer et al. used Dijkstra’s shortest path model to estimate the corona degradation of epoxy filled with micro/nano silica [94]. It was found that erosion path was longer in 62.5 wt% 16 µm + 2.5 wt% 25 nm silica filled epoxy in comparison to either only micro or nano filled composites. In another study, corona activities were performed for 20 min on epoxy filled with micro SiO_2_ (66 wt%) + nano TiO_2_ (1.6 wt%) and epoxy filled with only micro SiO_2_ (74 wt%) [95]. The results show that a micro/nano hybrid filler has superior performance as compared to its micro filled counterpart. The nano filler mixed at a low concentration into a micro filled composite gives a clear benefit in avoiding agglomeration as compared to using only micro or nano fillers at a higher concentration. This also suggests that the addition of a micro/nano hybrid filler is a favorable option to reduce the corona discharges as compared to adding either a nano or micro filled composite only.

### 3.5. Enhancement of Tracking and Erosion Resistance

When exposed to the field environment, polymeric insulators are prone to surface erosion and may ultimately form a permanent conducting path across the insulator. This phenomenon is termed tracking. During service, environmental contaminants and water droplets on the surface of an insulator are responsible for forming a conducting path allowing a leakage current to flow, which may cause surface erosion [96]. At present, no method is available to evaluate tracking and erosion resistance in the field. In a laboratory environment, however, two methods known as (1) the dust-and-fog test and (2) the liquid-contaminant inclined plane test as per the International Electrotechnical Commission (IEC) 60587 standard have been developed to evaluate the tracking and erosion resistance of a solid polymeric insulating material. The inclined plane test is more popularly adopted for materials used for high-voltage applications due to its severity than actual environmental conditions. This method is also easily reproducible to compare different materials. Using this method, various studies explored AC and DC tracking/erosion resistance of different polymeric materials. The parameters used for assessment included eroded mass, time verses track length performance and leakage current [97,98,99,100,101,102]. The inclined plane test when performed under DC voltage, a more degradation of polymeric materials in polluted environment was found. It is due to more heat generated due to consistent and unidirectional leakage current under DC voltage during dry band arcing and spot discharges at the insulators surface causing severe damage. 

In an attempt to improve resistance against tracking and erosion, various studies were conducted through incorporation of inorganic fillers in the base polymer and to evaluate their relevant performance. Nazir et al. [37] for example, reported that 30 wt% micro h-BN filled silicone rubber provides better resistance to AC tracking length, erosion depth and thermal distribution as compared to 30 wt% micro AlN-filled silicone rubber. Similarly, Boxue et al. reported that 40 wt% micro BN-filled epoxy composite inhibited thermal accumulation at discharge region which increased time to failure due to reduced tracking [103]. These results suggest that thermal accumulation was suppressed due to higher thermal conductivity offered by the filler material to the base polymer. 

Liu et al. [104] studied surface damage and bulk micro cracks of silicone rubber filled with nano silica after conducting inclined plane erosion tests. It was found that 1 wt% nano silica increases resistance to surface damage of silicone rubber and also reduces bulk cracks. Investigation on tracking failure of nano BN-filled EPDM was carried out using the inclined plane test. It was observed that 7 wt% nano BN takes the longest time before failure due to tracking takes place [105]. This behavior has been attributed to enhanced resistance to the formation of carbonized track which became possible due to thermal suppression ability of the added filler. Silicone rubber composites filled with different fillers like SiO_2_, Al_2_O_3_ and ATH were characterized to understand their effect on erosion resistance [106,107,108]. Overall, nano fillers perform better at lower concentrations as compared to their micron filled counterparts due to more restriction in polymer chain mobility at the interface of polymer and filler. This has been attributed to stronger interfacial bonding. 

The incorporation of micro/nano fillers into a polymeric material is one common effort to increase its resistance against tracking and erosion. For example, incorporation of micro and nano-sized BN particles in EPDM, the tracking time to failure increased by 42% in 15 wt% of 5 µm + 5 wt% of 50 nm as compared to 20 wt% micron filled counterpart [22]. Similarly, AlN and SiO_2_ fillers were added in silicone rubber with 27 wt% micro AlN + 3 wt% nano silica. This composite exhibited lower eroded mass (due to enhanced erosion resistance) in comparison to silicone rubber filled with 30 wt% micro AlN filled only [109]. The positive impact of enhanced tracking resistance of hybrid filler (27 wt% of ~5 µm AlN and 3 wt% of 20 nm SiO_2_) was linked to less temperature rise during dry band arcing due to improved thermal stability and thermal conductivity. Moreover, increase in the surface area of particles in micro/nano filled polymeric material results in better scattering and reduction of collision of secondary electrons.

## 4. Concerns of Electric Utilities and Way Forward

Insulator contamination has always been one of the major operational problems related to high-voltage transmission systems globally. Insulators installed in coastal and foggy areas as well as in different industrial and agricultural zones are exposed to a variety of pollutants, such as salts (soluble and poorly soluble), natural dust, coal, birds’ feces and flour [27,110]. These contaminants under high humidity offer a conductive layer on the insulator surface that facilitates the flow of leakage current [111]. Many researchers have conducted pollution studies on polymeric insulators made of SiR, EPDM and epoxy and analyzed different contaminants and their severity [112,113,114]. Three different artificial pollution test methods, namely the quantitative bushing method, the dipping method and the spraying method have been used to characterize the influence of salt deposit density on the AC flashover voltage [110]. In addition to that, the AC flashover performance and surface damage assessment of different polymeric materials in clean fog as well as salt-fog environments were investigated [115]. Moreover, the impact of pollutants such as salt fog, HNO_3_ and NaCl solutions on HTV-SiR has been studied [7,116,117]. Through this review, it has become obvious that new investigation techniques related to contamination studies [118,119,120] have not yet been implemented for smart composite insulators involving micro/nano fillers.

With proven performance and having a long life, ceramic insulators remained undisputed candidates for applications to insulate high-voltage transmission lines across the globe. However, with the rising level of transmission line voltages (to achieve better efficiency), the size of transmission infrastructure becomes large. Thus, the use of conventional ceramic insulators may not be the best choice. This becomes particularly more relevant where pollution conditions are severe, and lines can operate only with rigorous maintenance on a regular basis. The studies become more severe and dictating, particularly in those areas which experience heavy pollution, and the flashover probability of insulators increases. This prompted electric utilities and researchers to explore alternative means of providing insulation for high-voltage transmission lines.

The launch of the first generation of polymeric insulators made of silicone rubber, EPDM, and epoxy was the first major initiative in search of new insulators to replace conventional ceramic insulators. Relying on some obvious advantages of polymeric insulators, utilities around the globe welcomed these insulators and installed them on real systems. Among these, silicone rubber insulators showed superior performance and were readily accepted for more widespread use. The utilities were, however, aware of their organic nature and the tendency to age when exposed to environmental and electrical stresses in outdoor environments.

The electric utilities and researchers are in agreement that the life expectancy of polymeric insulators is the key issue which must be explored in full depth to come up with defendable recommendations which electric utilities can rely upon. It is in this context that new research initiatives look into the possibilities of using a composite material through the incorporation of anti-aging inorganic fillers in a base polymeric material. In recent years, research efforts have been made at the global level to use fillers of varying sizes and types and to evaluate the new composites in a variety of environments. Researchers are currently engaged in using different polymeric base materials and a variety of fillers to come up with the most reliable composite which should resist aging in a real environment and offer superior longevity. Through this review, it has become obvious that new investigation techniques related to contamination studies have not yet been implemented for composite insulators involving micro/nano fillers. Further, to develop new materials, a blend of SiR/EPDM with different modified nano fillers has become one of the areas of special focus [42,121,122].

## 5. Concluding Remarks

A detailed literature survey of polymeric materials used for manufacturing high-voltage transmission line insulators has been carried out. The first generation of polymeric insulators used silicone rubber, EPDM, and epoxy as shed materials, and through global experience, among them, silicone rubber was identified as the most promising material. To enhance the life expectancy of these insulators, research initiatives have been globally undertaken to incorporate micro, nano and mixed micro/nano inorganic fillers into the base polymer to retard the aging process. SiO_2_ is popularly used, which helps in bonding with silicone rubber and other fillers such as boron nitride (BN) to give thermal stability. The composite so formed enhances various properties, such as thermal conductivity, dielectric strength, mechanical strength, corona-discharge resistance, and tracking/erosion resistance. The selection of silicone rubber as the base material has been justified due to its ability to achieve self-recovery of its surface hydrophobicity. Through the incorporation of different inorganic fillers, researchers have been able to increase resistance against aging. Further work is still underway to find the most appropriate fillers and their optimum levels of loading to extend the life expectancy to a satisfactory level. As a result, the global manufacturers become able to adopt this technology to produce better products operating in a variety of global environments.

## Figures and Tables

**Table 1:** Polymeric materials used for outdoor high-voltage insulators and specified properties of fillers.

Ref.	Material	Thermal Conductivity (W/m·K)	Relative Permittivity	Density (g/cm^3^)
[37]	SiR	0.109	4	1.84
[38]	EPDM	0.25	3.3	1.53
[39]	Epoxy	0.17–0.21	3.6	1.16
[40]	Boron nitride (BN)	29–300	~4.3	2.25
[40]	Aluminium nitride (AlN)	~150–220	~9.2	3.26
[40]	Silicon nitride (Si_3_N_4_)	~86–120	~7.5–8.3	3.17
[40]	Silicon carbide (SiC)	~85	~6.5–10	3.21
[41]	Titanium oxide (TiO_2_)	~63.7	-	4.26
[41]	Aluminium oxide (Al_2_O_3_)	~38–42	~9	4
[42]	Aluminium trihydrate (ATH)	~25–40	-	2.42
[40]	Silicon dioxide (SiO_2_)	~3	~3.9	2.60

**Table 2:** Increased thermal conductivity of polymer matrix by adding different fillers.

Ref.	Fillers	Filler (s) Weight Fraction (%)	Filler (s) Volume Fraction (%)	Particle Size		Material			Thermal Conductivity (W/m·K)
				µm	nm	SiR	Epoxy	EPDM	
[44]	BN/AlN	16/17	64.5	5.5/1	-	-	√	-	12.3
[39]	AlN	80.8	60	7	-	-	√	-	11
[45]	BN	72	57	5–11	-	-	√	-	10.3
[46]	Al_2_O_3_/SiC	68/14	57	10/10	-	-	√	-	4.8
[40]	Al_2_O_3_	83.8	60	10	-	-	√	-	4.3
[47]	Si_3_N_4_	54	30	5	-	-	√	-	1.8
[48]	SiO_2_/BN	53/11	45	50/13	-	-	√	-	1.65
[49]	BN	50	34	15	-	-	√	-	0.84
[50]	BN	5	2.6	0.5	-	-	√	-	0.20
[43]	AlN	2	0.7	-	60	-	√	-	0.179
[43]	MgO	2.13	0.7	-	22	-	√	-	0.175
[51]	micro-Si_3_N_4_/nano-Al_2_O_3_	80/7	30	3	20	√	-	-	1.64
[42]	SiO_2_	50	41.4	10	-	√	-	-	0.97
[42]	ATH	50	43.2	5	-	√	-	-	0.75
[37]	BN	30	24.6	5	-	√	-	-	0.745
[52]	Al_2_O_3_/BN	20/20	30	1/10	-	√	-	-	0.64
[53]	BN	7	5.8	-	100	√	-	-	0.29
[37]	ATH	30	24.6	5	-	√	-	-	0.287
[37]	AlN	30	24.6	5	-	√	-	-	0.249
[54]	micro-BN/nano-BN	29/1	22.6	5	50	-	-	√	0.465

**Table 3:** Increased dielectric strength of polymer matrix by adding different fillers.

Ref.	Fillers	Filler (s) Weight Fraction (%)	Filler (s) Volume Fraction (%)	Particle Size		Material			Dielectric Strength (kV/mm)
				µm	nm	SiR	Epoxy	EPDM	
[61]	BN	10	5.41	-	70	-	√	-	230.6
[64]	Al_2_O_3_	5	1.50	-	7	-	√	-	212
[86]	micro-AlN/nano-AlN	20/3	9.6	10	50	-	√	-	168.1
[64]	Al_2_O_3_	60	30.32	10	-	-	√	-	90.3
[69]	Al_2_O_3_	1	0.3	-	40	-	√	-	59.9
[46]	Al_2_O_3_/BN	68/10	52	10/4	-	-	√	-	40
[68]	Al_2_O_3_	10	3.22	-	√	-	√	-	25.5
[68]	CaCO_3_	10	4.54	-	√	-	√	-	24.3
[68]	TiO_2_	10	2.93	-	√	-	√	-	19.3
[52]	Al_2_O_3_/BN	20/20	29.9	1/10	-	√	-	-	93
[51]	micro-Si_3_N_4_/nano-Al_2_O_3_	80/7	30	3	20	√	-	-	84.5
[58]	BN	12	8.6	-	√	√	-	-	25.5
[63]	micro-ATH/nano-SiO_2_	20/6	20.18	5	12	√	-	-	24.3
[62]	micro-Al_2_O_3_/nano-SiO_2_	20/4	17.38	-	-	√	-	-	23.9
[22]	micro-BN/nano-BN	17.5/2.5	26.5	5	50	-	-	√	89.2
[87]	SiO_2_	6	3.62	-	12	-	-	√	29.7

**Table 4:** Mechanical properties of SiR, epoxy and EPDM filled with micro and nano SiO_2_ fillers.

Sample	Ref.	Tensile Strength (MPa)			Elongation at Break (%)			Hardness (Shore A)		
		Virgin	Micro-Filled	Nano-Filled	Virgin	Micro-Filled	Nano-Filled	Virgin	Micro-Filled	Nano-Filled
SiR	[88]	0.5	0.9	1.6	142	123	118	48	54	56
Epoxy	[88]	1.2	1.6	2.7	125	115	110	54	56	58
EPDM	[88]	1.11	1.9	2.04	244.5	223.4	220.2	48.4	57.3	58.1

## Abbreviations

SiR	Silicone rubber
EPDM	Ethylene propylene diene monomer
SiO_2_	Silicon dioxide or silica
Al_2_O_3_	Aluminium oxide or alumina
BN	Boron nitride
EVA	Ethylene vinyl acetate
PTFE	Polytetrafluoroethylene
POE	Polyolefin elastomers
HDPE	High-density polyethylene
UV	Ultraviolet
TiO_2_	Titanium oxide
ZnO	Zinc oxide
MgO	Magnesium oxide
AlN	Aluminium nitride
Si_4_N_3_	Silicon nitride
SiC	Silicon carbide
ATH	Aluminium tri-hydrate
Ca_2_O_3_	Calcium carbonate
CB	Carbon black
AC	Alternating current
DC	Direct current
HTV-SiR	High temperature vulcanized silicone rubber
